# Editorial and Miscellaneous

**Published:** 1859-07

**Authors:** D. C. O’Keefe

**Affiliations:** New York


[Correspondence of the Atlanta Medical and Surgical Journal.]

New York, July 1st, 1859.

*Messrs. Editors*—On my way hither, I spent a few days in Philadelphia, with the view of examining its facilities for promoting the object of my visit North, which was to improve and extend my knowledge of medicine. There is no full regular course of lectures going on there during the summer months, since the dissolution of the Philadelphia Medical Association, and but few students or practitioners from the country are there at this time. Courses of lectures are delivered however, by private medical men, on most of the branches taught in our schools ; and among others, there is one on the use of the microscope as applied to the study of pathology, and one on diseases of the chest, including the physical signs, which I consider eminently useful and practical. The former course is given by Dr. Woodward, the latter by Dr. DaCosta. The college clinics are kept up twice a week, and are very valuable. These are continued during the summer, in order to keep the material on which they are based, from going elsewhere for treatment. Whether the city dispensaries afford any clinical facilities, I am not informed, but it is my impression they do not.

The chief hospital advantages exist in the Philadelphia and Blockley Hospitals. The Philadelphia Hospital, the first in importance to medical students, was founded in 1751, and relies for support mainly on private contributions and legacies. It, like many of the other benevolent institutions of the city, is supported for the most part, by the Society of Friends, and is devoted to the treatment of medical and surgical cases susceptible of deriving advantage therefrom—chronic diseases beyond the reach of treatment, are not admitted. All accidents, however, occurring in the State of Pennsylvania, if brought to the hospital within 24 hours after the occurrence, are admitted, and hence the amount of surgical material is quite abundant.

The average number of patients in the house for the year ending April 1858, was 162; whole number treated during same time, 1822, of which 1075 were surgical, and 747 medical cases. Of the acute medical cases most frequently seen at the south, there were of pleurisy 12 cases, pneumonia 12, dysentery 11, and typhoid fever 11. Thus of 747 medical cases, only 46 are of the forms of acute disease which constitute so large a proportion of the southern physician's practice. The privilege of attending this hospital costs $10, which allows admission only on the public days, twice a week, and then only in the amphitheatre, where the cases are brought in and lectured on. The privilege of going through the wards with the attending physician or surgeon, requires the additional recommendation of being a private student of these gentlemen, which entails an additional expense of $10 to $20. On the whole, I may say, that the advantages to the medical students, derivable from this institution, are considerably curtailed by the restrictions thrown around them.

The Blockley Hospital, also an Almshouse, is under the control of the city government, and contains a large amount of diseased material. Its 21 governors are elected annually, by popular vote, and they appoint the medical and surgical board. It is, therefore, a political institution, controled by the party in power; and its offices are sometimes conferred on favorites, in consideration for party services rendered.—While, therefore, its medical and surgical officers *may* possess talent, and the requisite qualifications, it is inevitable that unworthy persons are sometimes selected. A notable illustration of this fact, occured some time ago, in the selection of the notorious quack, McClintock, to a place on its medical board; but the event occasioned such a tumult in the profession, that the obnoxious act was soon annulled. The institution, however, possesses valuable advantages, which are accessible to all on payment of $5.

There may be other sources of clinical information in the city, but they were not brought to my notice—doubtless in the winter, they are more numerous, and made more available. Wills' ophthalmic hospital should not be omitted, in enumerating the medical advantages of Philadelphia. It was founded by the late James Wills, of the city, by a bequest of $122,584 for that purpose, and was opened for the reception of patients on the 3d March, 1834. The average number of inmates is about 40, but the number of "out patients" receiving attention from the institution, is much larger than the number of inmates. Since its foundation, upwards of 2000 persons have been cured or relieved, as inmates, and more than twice that number have received its benefits in their own houses. There are two weekly clinical days which are free to all.

I now come to the medical institutions of New York, and would premise that the limits I have assigned myself for this paper, will not permit me to give even an outline of them—I will merely allude to such as have come prominently before me.

The colleges are three, viz.: College of Physicians and Surgeons, founded in 1807; University Medical College, in 1841, and the New York Medical College, in 1850. The faculties of these colleges contain men well known to the profession throughout the country, and are eminently qualified for the discharge of their duties. There is a summer course delivered in each college, on subjects not embraced in the winter course—generally by junior members of the profession, not regularly connected with the colleges. Three or four clinics are held at each college weekly, embracing every department of medicine and surgery, and exceedingly interesting. There is a cessation of most of this college instruction, during the months of July and August, and a resumption of it on 1st September.

The hospitals of this city are numerous and extensive—25 in number—containing nearly 10,000 beds, and accommodating some 50,000 patients per annum. These, of course, are not all accessible to medical students, nor is it indeed, necessary. There are more of them accessible, however, than the medical student or practitioner can possibly find time to attend. The New York and Bellevue hospitals attract the principal attendance. The former was founded in 1771, by the Earl of Dunmore, at that time governor of the colony, and has accommodations for 350 patients. The U. S. Government send diseased seamen to this hospital, many of whom have contracted their sickness in warm latitudes, and hence a larger proportion of its medical cases belongs to the class of acute affections, prevalent in the southern States, than that of any other institution I have seen. Intermittent, remittent and typhoid fevers are well represented; so with rheumatism, pneumonia, pleurisy and diseased conditions of the liver. Its surgical wards, too, are richly supplied with material—most of the accidents, injuries, &c., of the city, are brought here, and hence the proportion of fractures and dislocations is large. There are clinics daily, and the admission is free.

Bellevue hospital, a department of the city almshouse, affords the most extensive opportunities of clinical study, of any other accessible institution in the city. The whole number of cases treated annually, is 7 to 8,000, of which a large proportion, belongs to the chronic incurable class.—Thus pthisis, delirium tremens, Bright's disease of the kidney, organic cardiac troubles, and every possible development of the broken down constitution, and every shade and variety of scrofulous diathesis, all and singular, find here a full representation. So that if the morals and sanitary condition of the city were viewed from this stand point *alone*, the impression would be an unfavorable one.

At the Dead House of the hospital, autopsies are made almost every day, thus affording excellent facilities for the study of pathology. The most popular clinical instructor at the hospital at this time, is Dr. Metcalf, Prof. of Practice in University Medical College. He has public (bed side) clinics twice a week in the medical department, which are largely attended. Dr. M. is comparatively a young man, about 40, I should think, a native of Natchez, Miss.; educated at West Point, he served some time in the army, and participated in some active service, I think, in the Florida war. Tiring of military glory, he selected the peaceful field of medicine for his profession, and now stands *inter primas* as a teacher and general practitioner.

I have dwelt somewhat minutely on this gentleman, because he has impressed me as being a man of extraordinary talents and accomplishments. His examination of a patient presents before the mind a well-defined picture of the case, in all its relations and bearings.

Dr. Clark, Prof. Practice in College cf Physicians and Surgeons, is also an excellent clinical teacher, whose service at Bellevue is well attended. There is a difference of opinion as to which of these gentlemen is the better practitioner and instructor, and each has his admirers. Dr. C. is, in my opinion, quite as efficient as a teacher, and perhaps a more experienced pathologist, and, as I said, some prefer him to Dr. M.; but there is so much of the *suaviter in modo*, combined with sound judgment and ready perception in Dr. M., that he is considered a more popular teacher. The fee for attendance at Bellevue is $5.

On the three Islands of Blackwell, Ward & Randall, are vast hospital institutions which are not used for clinical instruction, on account, I suppose, of their inaccessibility, and the fact, moreover, that there are more facilities in the city than one has time to avail himself of. Every Saturday, however, Dr. Carnochan, the eminent Prof. Surgery in N. Y. Med. College, holds a valuable surgical clinic at the Emigrant's Hospital, Ward's Island.

The Eye and Ear, Nursery and Child's Hospitals, afford valuable facilities for studying their respective diseases, and have clinical days several times a week. The eight Dispensaries, too, afford unlimited opportunities of seeing every possible variety of disease, medical and surgical, and the medical gentlemen in charge of them afford the medical enquirer every facility of improvement—five hours a day could be profitably spent in some of these institutions.

The Pathological Society, and the Academy of Medicine meet each twice a month; so there is a meeting of one or the other every week. They are valuable sources of knowledge, sustained by the first medical men in the city. At the last regular meeting of the former, I had an opportnnity of examining nine recent pathological specimens of great interest, and hearing the history of the cures from which they were obtained; and this is about a fair sample of the objects of interest presented at its meetings. At the Acad. Med., they are discussing at this time, the subject of the personal communicability of Yellow Fever, and are having an interesting time of it.

Last, though not least, I must invite your attention to the Woman's Hospital, over which presides the talented and accomplished southerner, Dr. J. Marion Sims, formerly of Montgomery, Ala. Unable to live at the south, on account of a chronic diarrhœa, he became a resident of this city in the fall of 1853, not of choice, but of necessity. His reputation in the cure of that intractable misfortune of woman, vesico-vaginal fistula, soon brought him patronage and position, and was instrumental in founding the Woman's Hospital, which was open for the reception of patients on the 4th May, 1855—less than two years after his residence here. The accommodations at present are limited, and not at all commensurate with the demands made npon it—its prospects for the future, however, are flattering. With a fund, of about $60,000 on hand, and grounds obtained from the city, said to be worth $100.000, and an implied understanding in its charter, of obtaining from the State a large appropriation, it is proposed to found a Woman's Hospital with 200 beds, devoted exclusively to the treatment of diseases peculiar to women, which will be an honor not only to the city and State of N. Y., but to the whole country.—The hospital is not confined exclusively to the management of fistulous cases, but extends its benefits to all the derangements of the generative organs. Dr. Sims does an extensive private practice in the same class of cases; he does not leave his office to see a case; does not prescribe for man or child, or woman, unless her ailment comes within his range; he is, therefore, peculiarly a woman's man. A full history is taken of every case, and if of sufficient interest, an original drawing is made of the diseased condition by his accomplished assistant surgeon, Dr. Thomas Addis Emmet. This hospital has no public clinics, but its operations are witnessed by many medical men, from various parts of the country, who call upon Dr. Sims—he has had the kindness to tender your correspondent a standing invitation to visit it at his pleasure, and I have already seen much that I deem of great interest and value.

As regards the comparative merits of New York and Philadelphia in their facilities for imparting a medical education, I think there is an erroneous public sentiment prevalent at the south. For my own part, I consider the clinical advantages of New York eminently superior to those of Philadelphia; in fact, they are practically illimitable. A man who reads not a line cannot attend the half of them, simply for want of time. The teaching faculties of Philadelphia, may be more eminent—may have written more books—may occupy more prominent positions before the medical mind of the country, but such a state of things cannot last. If the profession of this city be true to itself; if it continue to cultivate and encourage the zeal and talent it possesses, it cannot fail to reach a metropolitan position before the country.

I intended saying something about the medical *celebrities* of this city, but must defer it to another time.

Truly yours,

D. C. O'KEEFE.

